# Relating total hip replacement and acetabular cup positioning with outcome: A systematic review

**DOI:** 10.6026/973206300210145

**Published:** 2025-02-28

**Authors:** Ankush Mohabey, Prajakta Warjukar

**Affiliations:** 1 Department of Orthopaedics, All India Institute of Medical Sciences, Maharashtra, Nagpur, India; 2 Department of Biochemistry, Datta Meghe Medical College, Nagpur, Datta Meghe Institute of Health Education and Research (DMIHER), Sawangi, Wardha, Maharashtra, India

**Keywords:** Total hip replacement, dislocation, revision rate, offset, acetabular cup, femoral head, infections

## Abstract

The diverse methodologies employed in assessing cup placement, delineate the recommended target zones for positioning and examine the correlation between cup positioning and occurrences of complications is of interest. We included 51,308 patients and 51,692 hips for this analysis. The overall complication rate was 22.2%. Patients, overall, demonstrated improved outcomes, as evidenced by postoperative hip scores. Two "safe windows" have been defined for surgical procedures: 1) an inclination of 35-50 degrees and an anteversion of 5-25 degrees and 2) an inclination of 35-50 degrees and an anteversion of 15-25 degrees.

## Background:

One of the most effective surgical techniques is total hip replacement (THR), which has been called the "operation of the century." Worldwide, over a million surgeries are carried out every year and within the next ten years; this number is expected to triple [1]. Cup position is affected by a number of factors, including the structure of the pelvis, the stiffness of the spinopelvic junction, the functional positions of the pelvis, its position during configuration and motion during surgery, the use of reference frames, the method used to quantify angles and surgical skill [2]. The idea of a safe zone for acetabular component orientation based on CT show that the previous Lewinnek secure zone is not a good indicator of stability in the future [3]. THR provides notable improvements in pain, mobility and physical function along with outstanding technical outcomes, with a 10-year survival rate of over 95% and a 25-year implant survival rate of over 80%. However, the patient's experience following surgery or attempts to receive care may not be reflected in these conventional measures of surgical success [4]. In recent years, patients undergoing total hip replacement (THR) increasingly anticipate maintaining active lifestyles long after their surgeries. This shift in expectations has heightened focus on health-related quality of life (HRQOL) and patient-reported outcomes. Studies have shown that while many patients expect significant improvements in mobility and daily activities post-THR, some experience challenges in meeting these expectations, underscoring the importance of preoperative discussions to align surgical outcomes with patient anticipations [5]. The ageing population and the superior results of total hip arthroplasty (THA) surgery will lead to a rise in the number of total hip arthroplasty procedures. Successful long-term fixation requires adequate fixing of the uncemented acetabular component in order to produce bone in-growth and on-growth [6]. While press-fit cement less cups is effective in achieving initial stability, inadequate fixation can result in cup migration and reduced survival rates. Studies have shown that insufficient press-fit fixation may lead to implant loosening during or after surgery, adversely affecting long-term outcomes. In such cases, supplemental screw fixation can enhance cup stability; however, the benefits and limitations of both press-fit and screw fixation techniques should be carefully considered to optimize initial fixation and long-term success [7]. Thus, the problem of the acetabular cup migrating and becoming loose, requiring revision surgery remains unresolved. To increase the stability of the acetabular cup after initial fixation, additional screw fixation is frequently used [8]. There have been cases of peri acetabular osteolysis due to the joint fluid and polyethene wear particles infiltrating through the screw hole, which may eventually cause the cup to loosen, even though it may not lessen the need for revision or reoperation in the future. As a result, it is still unknown if screws in acetabular cup total hip arthroplasty are long-term beneficial [9, 10]. Considerable methodical reviews and RCTs have examined the effectiveness of screw fixation in cement-less cup total hip arthroplasty as well as assessments of cup positioning, dislocations and placements. Therefore, it is of interest evaluate the existing RCTs that looked into the measures of cup positions, dislocations, targeted cup placements, anteversion and inclination angles and their revision rate and offsets using known data.

## Materials and Methods:

The "International Prospective Register of Systematic Reviews ID (PROSPERO ID)" of the study is CRD42025646319. The Preferred Reporting Items for Systematic Reviews and Meta-Analysis (PRISMA) statement was strictly followed in the conduct of a thorough systematic review and stringent inclusion criteria were maintained at all times. Thoroughly recording and examining the reported occurrences of postoperative dislocations was one of the primary goals of this investigation. Furthermore, the cup location measurements, which comprised anteversion and inclination angles, were carefully documented and assessed. To guarantee correct assessment, the target zone of cup placements was accurately specified. In order to offer a detailed comparison of the surgical techniques, the study also looked closely at the revision rate, Offset and functional outcomes.

## Search strategy:

A thorough and in-depth examination of the PubMed database was necessary in order to find pertinent publications. The search was limited to English-language works that had been released in the previous seven years in order to include the most recent and relevant research. It's also important to note that web searches and Google Scholar were purposefully left off the list of sources for unpublished material.

## Inclusion and exclusion criteria:

Our study included adult human individuals (above the age of 18) who had undergone primary total hip arthroplasty (THA) and did not have any prior anatomical, structural, or metabolic problems (such as osteoporosis, storage disorders, or malignancies) that could compromise the integrity of their bone. In our analysis, we included any research, regardless of study type, that used a head diameter of 28 mm or more. Research on congenital hip problems such as Developmental Dysplasia of the Hip (DDH), Achondroplasia, dwarfism or gigantism was included from the analysis. The papers we included were only concerning primary arthroplasty. These also included dislocation surgeries done on patients at high risk. Every English-language publication from 2018 to 2024 that includes relevant patient data and has a minimum one-year follow-up time has been included. Research that only showed effects in a simulated environment or in animal or laboratory settings has been disqualified because of their inherent clinical limitations.

## Data extraction:

The primary objective of the research was to investigate the relationship between the acetabular cups and femoral head sizes and how they affect better functional outcomes. To determine the link between these factors, data from other research had to be gathered and analyzed. In addition, the study carefully recorded the attributes of the studies it included and the individuals who took part, including information about age, gender and any pertinent medical history. The study also examined particular recurrent subjects of conversation, including revision rates, dislocation incidents, offset measures and prevalent illnesses. A detailed description of the findings was provided to give a complete picture of the research results.

## Results:

## Screening:

## Study selection:

We methodically prepared a list of the titles and abstracts of pertinent articles. Complete papers were collected for in-depth analysis in cases where conclusions could not be drawn from the abstracts. Examining references in detail made it easier to access grey literature and reduce errors in electronic database searches. In order to guarantee comprehensiveness, cross-referencing citations in review papers was also done. Based on pre-set inclusion criteria, titles and abstracts were independently screened. Each potentially eligible study was then paired with its corresponding full article, which was then painstakingly classified with a distinct identity for organized tracking and expedited identification in the stages that followed. To ensure review precision, we systematically excluded research that required more adequate data, was not accessible via electronic methods, or presented irrelevant information (Figure 1 - see PDF).

## Demographic data:

Initially, 51,308 patients and 51,692 hips were included in the analysis. However, only 18,081 hips made it into the final analysis after 22,332 hips were eliminated owing to incomplete data or loss of follow-up. At the time of surgery, these hips in age from 59 to 79 years, within age of 70. These hips had follow-ups every 4.3 years on average, with intervals of 1.3 to 11 years.

## Measurement of cup positioning:

Recently, [11] described the anatomical, radiographic and surgical methods for assessing angles of anteversion and inclination. Standard anteroposterior (AP) radiographs can be used to determine radiographic anteversion and inclination. As seen in Figure 2a (see PDF), cup inclination is generated by the plane of the acetabular aperture and the transverse axis, or ischial tuberosity line. Figure 2b (see PDF) provides an explanation of how cup anteversion is calculated [11]. AP radiographs are not reliable in reliably differentiating between cup retroversion and anteversion; lateral radiographs are necessary for calculating version of cup in cases where CT scan is not feasible [12]. By recognizing the angle between the transverse axis and the acetabular opening on a shoot-through lateral radiograph, as seen in Figure 2c (see PDF), one can compute cup anteversion. When compared with the AP radiography computation, this method tends to overstate anteversion. This methodology was applied in one study [13]. The most popular and practically feasible way to determine cup location is through standardized AP radiography; however, this method is not accurate when compared to anatomical anteversion because it does not take pelvic positioning or spine deformity into account.

An accurate measurement of anatomical anteversion and inclination angles is made possible by computed tomography (CT) (Figure 3 - see PDF), which provides a three-dimensional evaluation of acetabular cup location. The angle between the acetabular axis' projection onto the transverse plane and the left-right axis is known as anteversion, while the angle between the acetabular axis and the coronal plane is known as inclination [14]. The established confounding factor in radiological measurements, pelvic placement, has no effect on these angles. Cup anteversion was determined via CT analysis in 4 investigations [15, 16, 17-18]. One of two methods was employed to calculate inclination: additional AP radiographs or AP radiographic reconstructions from CT. Two studies [19, 20] did not specify the techniques used to measure cup anteversion and inclination. A goniometer and computer-assisted surgery (CAS) were the intraoperative instruments that were used in two investigations to help with the placement of the acetabular component.

## Dislocation:

According to the body of current research, head size and dislocation are inversely correlated as long as the implant is positioned correctly. Based on long-term follow-up investigations in high-volume and combined registry studies, our data firmly confirm this finding. Seven studies reported 0% to 1% to 5% (Table 2); however, these studies had tiny sample sizes [21, 22]. The risk of dislocation is the same for posterior or postero-lateral approaches as for other methods; however, the risk of dislocation is higher for Minimally Invasive Surgery (MIS) procedures, independent of approach or head size. Greater head size can improve stability, but only if the cup is oriented correctly [23]. If the cup is positioned incorrectly, the benefit of stability is lost and the chances of dislocation increases. Large heads (**36 mm), dual mobility liners and restricted acetabular liners are alternatives for minimizing future dislocation [24]. When stratified by surgical method, the overall 6-year revision rate for dislocation was 0.5-0.6%. Since there are several contributing factors to hip instability, restoring the femur and acetabulum's Offset separately or together, preserving soft tissue balance and having a large head diameter all help to stabilize the hip and diminish the chance of dislocation [25-26]. We consider it impossible to determine at this time if a big femoral head alone lowers the dislocation rate. It reduces, if not completely eliminates, the danger of hip dislocation and, at most, increases hip stability. The [27] found that the survivorship rate for dislocation at ten years was 95.6% and at 20 years, it was 90.6%. The 10-year survival rate for any event was 89.4% and at 20 years, it was 82.5%. Just over 7% of patients experienced a dislocation of their constrained liners. 4.4% of hips experienced a deep infection. Additionally, 1.8% of patients had dissociated constraining rings and Periprosthetic femoral fractures.

## Anteversion and inclination angle:

The distribution of angle of inclination and cup anteversion between hips that displaced and those that did not was calculated and compared in 19 of the 28 articles [28, 29, 30-31]. Only inclination was examined by the author. Three out of the nineteen trials found a statistically significant variance in the mean inclination values between hips that were dislocating and those that were not. It was shown that anteriorly displaced hips were more extended than posteriorly dislocated hips (anterolateral approach). These findings were in contrast to hips that did not dislocate. Hips with posterior dislocation were found by the [32] (posterolateral approach) to have larger inclination angles than hips that were not dislocating. According to the [32] (posterolateral) research, hips that dislocated generally had larger angles of inclination than hips that did not. Mean anteversion angles were observed to differ statistically substantially between non-dislocating and dislocating hips in 6 out of 8 investigations [33, 34, 35-36]. Three of these investigations employed the anterolateral, one the posterior and four the posterolateral approaches. Four studies looked at anterior and posterior dislocations independently; three of them concluded that anterior dislocations were linked to higher anteversion, whereas the other two concluded that posterior dislocations were less anteverted than non-dislocators. Based on the author's research, hips that dislocated posteriorly had less anteversion than hips that did not. One study [36] indicated that the dislocations were less anteverted in this instance, but it did not specify if they were anterior or posterior. Significant heterogeneity was found when mean angles of anteversion and inclination were examined amongst articles. The majority of the papers failed to find statistically significant differences in mean anteversion values (12/18) or mean inclination values (16/19) between dislocating as well as non-dislocating this.

## The target zone for cup placement:

The author suggested a safe zone with a 30 to 50-degree inclination and 5 to 25-degree anteversion as a way to reduce postoperative dislocation. The [37] suggested that an inclination range of 30 to 45 degrees was more optimal, given the correlation between excessive inclination and an increased rate of wear and edge loading. 16 experiments discovered a range of anteversion and inclination angles that may be used to securely position the acetabular component; these new target zones differed from the range of values that the author had originally recommended [38, 39- 40]. A statistically substantial reduction in the possibility of dislocation was seen by the author (Tran's gluteal approach) for inclinations between 35 and 55 degrees and anteversion between 5 and 25 degrees. For this target range, the author (76% anterolateral THA) did not find any differences. They measured anteversion ranging from 10 to 30 degrees for the posterolateral approach and discovered a statistically significant decrease in dislocation risk. Three investigations with varying target ranges of anteversion and inclination reported a statistically significant decrease in cup dislocation which are as follows: [41] found that 30-50 degrees of inclination and 10-25 degrees of anteversion, the [42] found that 35-55 degrees of inclination and 5-25 degrees of anteversion, while [23] found that 27-57 degrees of inclination and -3 to 27 degrees of anteversion has less chances of dislocation. There are two "safe windows" that [43] identified: (1) 5-50 degrees of inclination and 5-25 degrees of anteversion and (2) 35-50 degrees of inclination and 15-25 degrees of anteversion *i.e.* [43] reported posterolateral surgery; [41] reported posterior surgery; [23] reported 74% anterolateral surgery; and [44] reported anterolateral surgery. Severe outliers of cup positioning from the target zone have a more increased risk of dislocation, even though [23] target range was wider (± 15 degrees) than the other ranges discussed above. Eleven investigations juxtaposed the aggregated anteversion/inclination values with their positioning within or beyond the secure area delineated by [45]. Placing cups in the Lewinnek safe zone reduced postoperative dislocation statistically significantly, according to only two of these investigations [46]. This was compared to the four articles [47, 48, 49-50] that found no significant difference between dislocating and non-dislocating hips. Four investigations [47, 48, 49-50] revealed that there were more hip dislocations outside than inside the Lewinnek safe zone. According to seven investigations [47, 48, 49, 50, 51, 52-53], there were more dislocating THAs found in the Lewinnek safe zone than outside of it. A study by [53] found that 83 per cent of dislocating hips were in the safe zone, compared to 53 per cent of non-dislocating hips. In terms of positioning and dislocation rate, [54] compared the acetabular component's placement when it was navigated and when it wasn't. In the Lewinnek safe zone, 81% of cups placed using the navigated approach landed, compared to 63% in the non-navigated group (p < 0.001). Additionally, the navigated group experienced 8/779) fewer dislocations than the non-navigated group (17/684) (p = 0.03). The two groups' mean anteversion and inclination angles were comparable [54].

## Infections and other complications:

Out of 3598 hips, 5.2% had implant aseptic loosening, either for the femoral stem or the cup. The aseptic loosening of the stem resulted in a 1.5% reoperation rate (49 of 3345 hips), while the aseptic loosening of the cup caused 2.9% (103 of 3598 hips) of reoperations. 4.6% of hips (153 out of 3345) had an infection overall; 2.9% of those hips had a deep infection that required two stages of revision total hip arthroplasty (THA); the remaining hips had an acute infection that required debridement, antibiotics and implant retention. Surgery was used to treat every infection, with an overall 4.6% of hip reoperations being related to infection (153 out of 3345 hips). Of 3345 hip replacements, the incidence of hematoma, seroma and wound complications was 0.5%. An uncommon consequence that was recorded in 0.1% of instances (4 of 3345 hips) was nerve damage. 3.4% of cases (111 of 3304 hips) had additional complications, such as Periprosthetic fractures, implant failure and component breakage; 2.2% of cases (74 of 3304 hips) required reoperation. Out of 578 hips, the total incidence of infection was 7.6%. Of those, 44 hips required further reoperation in 84.0% of cases and 7 hips received conservative treatment with suppressive antibiotic medication in 15.9% of cases. Of the 37 cases (62.2%) of infected hips that needed surgery, revision or resection, arthroplasty was done in 23 and debridement, antibiotics and implant retention (DAIR) was done in 14 cases (37.9%) of infected hips. Of the 578 hips, the total reoperation rate as a result of infection was 6.4%. Of the 578 cases, 41 instances (or 7.1%) had additional problems noted. Of those, 19 out of 41 problems required surgical treatment in 46.3% of the patients; the most common complication requiring additional surgery was Periprosthetic fracture (12 hips, 2.1%). Revision of the implant was recorded in 75% of instances (9 out of 12 hips) and osteosynthesis in 25% (3 hips) of cases with Periprosthetic fractures. 53.7% of the 41 problems did not require surgery, with DVT being the most common, with a reported incidence of 1.2% (7 hips).

## Functional outcome:

Following surgery, the Hip Disability and Osteoarthritis Outcome Score (HHS) ratings showed a consistent and noteworthy improvement across all investigations. There was a high degree of variation among the research, as indicated by the heterogeneity index (I2) of 97%. With a 95% confidence interval of 36.48 to 47.86 points (p<0.001), the HHS scores showed a significant average improvement of 42.17 points out of 100.

## Revision rate:

Prior research revealed that younger patients had higher revision rates after Charnley low-friction arthroplasty than older cohorts did. The primary causes of failure were wear-induced osteolysis and aseptic loosening. Five studies that report the total revision rate in larger sample sizes with a revision risk ranging from 1 to 5% were identified (Table 2). Because of ARMD (adverse reaction to metal debris) problems, the metal on metal (Metal on metal) combination had the highest reported revision rate (7.5%). According to the Swedish registry study, female sex, posterior approach, MIS, small head size (RR=2.5, CI 1.1-5.7; p=0.03) and posterior method are related to an increased risk of revision factors. Comparing LDH metal on metal total hip arthroplasty to cemented total hip arthroplasty another Finnish registry study reveals that revision rates were higher in female sex patients 55 years of age or older and that revision rates were not statistically significantly impacted by head diameter or diagnosis. An annualized revision rate of 0.6% was obtained from the 37 hips (5.0%) that were revised after an 8.4-year weighted mean follow-up among the included studies for review.

## Offset:

## Femoral offset:

First, within the offset parameters, this review will deal with the femoral component. Several studies have assessed the advantages and disadvantages of varying the femoral Offset. Previous research [55] has typically been critical of reducing femoral Offset. One of the first notable papers on this subject was written by [56]. Who discovered that patients whose original femoral offset was reduced by more than 5 mm had worse functional outcome scores than patients in the groups with patched and increased offsets? Reduced femoral Offset has been shown to modify gait, which is consistent with the findings of [57-58] discovered that there was no correlation between a reduced femoral offset and a higher risk of dislocation [59]. Have conducted a study of simulation results related to capacity and have determined that, in line with Pauwels' biomechanical model of the hip a reduced femoral offset results in an increase in abductor muscle power and hip joint connection points to maintain the hip abduction moment. Accordingly, every one of these research suggests that femoral Offset shouldn't be decreased following surgery. That being said, not every member of the scientific community agrees with every other member [60].

## Acetabular offset:

Compared to the literature on femoral Offset, less research has been published on the topic of acetabular Offset. As a matter of fact, the literature on acetabular Offset has become less active and produced fewer papers [61]. A recent study [62] found that although both femoral and acetabular offsets are thought to be significant for restoring hip joint architecture, the majority of research has concentrated on femoral Offset in relation to gait and function. According to an early study [63] on this topic, for the best acetabular component coverage, the original hip centre of rotation should be restored or slightly medialized. In reality, this idea demonstrated better functional results when combined with a higher femoral offset that compensates for it. According to [64], medialization resulted in a considerable diminution in acetabular Offset (5 mm) with no change in global Offset, corresponding with a significant rise in femoral Offset (5 mm). The proposed scientific rationale, as presented by [65], is as follows: A decrease in acetabular offset might produce a positive impact by reducing the stress utilized to the prosthetic joint and would need counterbalancing by boosting the femoral offset to avoid decreased the global offset which would needed limb elongation to avoid instabilities.

## Global offset:

Studies nevertheless emphasize that global Offset-the whole sum of the femoral and acetabular components is a crucial component to take into account when analysing its subgroups within the offset domain [66]. Emphasize that the global Offset is a more dependable joint parameter than femoral Offset alone for restoring hip parameters following primary total hip arthroplasty (THA) since it accounts for both acetabular and femoral Offset. However, since acetabular and femoral offsets can be adjusted separately, this begs the question of whether the resulting global Offset should be retained, increased, or lowered overall. Many researchers have looked into and criticized the decrease in global Offset, which is sometimes referred to as a surgical error [67]. For example, a warning was issued that decreasing global Offset by more than 5 mm is associated with worse functional outcomes for patients. In fact, when global Offset was sufficiently reinstated according to reduced acetabular Offset rendering to Pauwels's definition and increased femoral Offset, [68] demonstrated enhancements in gait pattern, + discomfort and health-related quality of life. It is noteworthy that Clement and colleagues also demonstrated that enhanced functional results are obtained with a large reduction in acetabular Offset, an increase in femoral Offset and no overall significant alteration in global Offset. Various offsets- outline of crucial consequences from the literature was noted and tabulated (Table 1).

## Discussion:

We examined eight included studies in this systematic review in order to measure cup positioning, assess the impact of cup mal-positioning on dislocation rates after primary total hip arthroplasty and pinpoint suggested target zones for cup anteversion and inclination to lower the risk of dislocation. The bulk of issues with the assessment and comparison of articles had to do with the methodology and design of the studies. The cup version of one study could only be categorized as retro-, normo-, or anteverted, which precluded a statistical comparison with the other papers. Studies [83, 84, 85, 86, 87, 88, 89, 90, 91, 92- 93] that looked at cup location radiographically did not mention using lateral pelvic radiographs to distinguish between anteversion and retroversion [94]. Said that anteversion was assumed. According to other papers, hip anteversion calculations were never done in the absence of a lateral radiograph. Cases of hip dysplasia were included in five studies' samples that were examined for cup placement versus dislocation. This could have had an impact on the suitability of the angle ranges found in the context of non-dysplastic hip arthroplasty since hip dysplasia has been demonstrated to manipulate cup location recently. Only one study reported a dysplastic hip total hip arthroplasty rate of more than 5%. Three studies that did not distinguish between cases of primary and revision arthroplasty in their analysis had a similar problem. It should be noted that 6 out of 9 dislocations in the study were revision instances [95]. Although the author determined the percentage of all instances that were primary total hip arthroplasty neither the dislocation nor comparison groups received this information [96]. Dislocation rate and cup positioning angles are included in this study's listing of primary and revision THAs since primary THAs could not be distinguished from the sample given. Since one study's data showed an indirect comparison between cup positioning and dislocation rate, it was not included in the comparison with the other papers [97]. We considered the results of that study to be pertinent to our research issue and included them for qualitative reasons, even though it included a potential confounding variable. The lack of adequate statistical power was also mentioned as a potential barrier to validating and comparing the results of articles. The size of the study groups varied greatly throughout publications; [98] whose research has low statistical power would find it challenging to replicate their findings with higher sample numbers. It was not possible to evaluate the research in this way since it did not provide the extent of the patient sampling from which the study groups were selected [98]. When measuring anteversion/inclination angles, ordinary radiographs give definitions of anteversion and inclination that differ from CT's anatomical definitions. In certain research, computer processing was utilized to compute anteversion and inclination angles. Comparing cup positioning angles and target zones across research was thought to be complicated by the fact that different articles used different manual methods for calculating angles. Because of this, we advise measuring anteversion and inclination angles using a uniform methodology. The benefits of using CT include measurements that are unaffected by pelvic positioning, more accurate identification of angles (especially anteversion) and the ability to calculate femoral version. These findings have been documented by [99, 100]. Nevertheless, it seems that the most straightforward and economical approach to date is computer analysis of conventional AP and lateral radiographs. Moreover, functional cup positioning in the standing position is represented by acetabular cup positioning, which is assessed on standardized standing AP radiographs and ought to be advised. This contrasts with the anatomical position determined by CT scanning, as well as the potential for altered positioning as determined by supine AP and shoot-through pictures [101].

According to [102], the ideal site for lowering the risk of dislocation may not always coincide with the target zone, which lowers the risk of other problems such as wear, impingement and revision rates. It has been demonstrated that additional elements like pelvic tilt and component sizing affect the risk of dislocation and the proper alignment [103]. As a possible factor in hip stability, the femoral version and a combination of the acetabular-femoral version have also been studied [104]. These factors, however, were not examined because they were outside the purview of this review. The probability of anterior and posterior dislocation can be influenced by the surgical strategy used; in certain cases, a different target zone may be advised [105]. For instance, a posterior approach would increase the risk of posterior dislocation by causing soft tissue and muscle weakening at the operative site. According to the hip joint's mechanics, increasing anteversion in this situation should potentially lessen the likelihood of a posterior dislocation [106]. Lower anteversion angles were recorded in several trials included in this analysis when dislocating THAs using the posterolateral technique. This may imply the requirement for target zones unique to the total hip arthroplasty surgical technique. Of these, two articles [105] [106] identified a statistically significant difference, whereas [107, 108, 109-110] found no statistically significant reduction in hip dislocation for cups placed in this zone. Five investigations were able to determine the anteversion and inclination target zones that minimized the chance of dislocation. Four papers, in contrast, revealed target zones with an identical dislocation rate. According to two of these research [111, 112], cup anteversion has a rather narrow range of 10-15 degrees. Small sample sizes may have hampered the other two, even though they tested a wider range of targets [113]. This may imply that these target zones need to be reinterpreted in some situations or that the suggested target zones ought to be increased due to their impracticality. The 5% revision rate at 8.4 years is comparable to the general total hip arthroplasty population. This suggests improved techniques and implants in recent literature. As an alternative, new technology designed to support precise cup placement may make it possible to replicate small target ranges in a clinical setting. Compared to freehand methods, the use of CT-based or imageless navigation systems has been linked to improved placement in a designated target zone and reduced variability in cup placement [114]. In order to more accurately assess the proper cup location for each patient, these techniques can make use of patient-anatomical landmarks such as the pubic tubercle and anterior superior iliac spines [115].

In conclusion, our comprehensive analysis of pertinent literature revealed that while some studies were unable to find a correlation, others demonstrated that cup positioning had an impact on postoperative dislocation. The majority of the publications did not detect a statistically important distinction between the groups of dislocating and non-dislocating THAs when comparing the mean angles of anteversion and inclination. It is challenging to generalize the goal zone for cup location in total hip arthroplasty because of the diversity of study methods, surgical techniques and patient demographics that have been discovered. There are likely a number of other factors that affect the target zone for cup placement. Therefore, each patient's optimum target zone may differ based on these circumstances. While it might not completely eliminate the danger of dislocation, positioning the cup in a target zone could help to reduce it. Future research examining the positioning of the acetabular cup and the risk of dislocation ought to evaluate other surgical techniques independently, as these techniques could impact the ideal placement of the acetabular component.

## Conclusion:

Data shows that the average anteversion and inclination angles between hips that dislocated and those that did not were not significantly different. It is difficult to definitively identify the ideal target zone for cup positioning in total hip arthroplasty (THA) because of the variety of study methods, surgical techniques and patient demographics taken into account. Two "safe windows" have been defined for surgical procedures: 1) an inclination of 35-50 degrees and an anteversion of 5-25 degrees and 2) an inclination of 35-50 degrees and an anteversion of 15-25 degrees. Other factors also influence the ideal cup placement, resulting in variation based on individual patient characteristics. Patient over 18 years old shows reliable outcomes for up to 10 years post-THR. Modular designs have reduced dislocation rates.

## Future recommendations:

Future research on the positioning of the acetabular cup and the risk of dislocation should examine other surgical techniques, patient characteristics and use of advanced techniques and prosthesis.

## Figures and Tables

**Table 1:** Various offsets- outline of crucial consequences from the literature

**References**	**year**	**Cases**	**Study type**	**Offset**	**Parameter related to Offset**	**Key findimg**
[69]	2021	18 Patients	Prospective	Ipsilateral Offset		Increased abductor moment arms were obtained with a 2.3-2.9 mm increase in FO, while the maximum contraction of the hip muscles was kept below5.0%.
[70]	2021	26 normal hips	Simulation	Ipsilateral Offset	Simulated acetabular component coverage rate, micromotion,and peak stress distribution	The anatomical approach has no stress concentration and less micromotion than the al technique, which has higher coverage rates.
[71]	2021	131 Hip OA	Simulation	Ipsilateral Offset	NA	Significant differences in natural acetabular offset between individuals (up to 13 mm). There is a chance of up to 19 mm of excessive medialization when reaming down to the actual floor of the acetabulum.
[72]	2020	121 patients	Prospective	Contralateral Offset	Simulated hip range of motion before impingement	Simulated under restoration reduced the number of patients meeting the ROM requirements for ADL by more than 20% in patients with highstems. Simulated over-restoration of Offset causes a modest rise of less than 10% in patients with standard Offset stems.
[73]	2021	91 patients	Prospective	Preoperative ipsilateral Offset	Gait speed	The postoperative gait speed was not significantly influenced by global offset.
[74]	2021	65 patients	Prospective	Preoperative ipsilateral Offset and contralateral Offset	HOOS, EQ-5D, gait analysis	With restored global offset based on increased femoral offset and medialized acetabular offset, gait pattern and discomfort improved. An increase in walking speed and an upright posture were associated with the higher hip adduction moment, rather than a shift in offset quota.

**Table 2:** Characteristics of included studies

**References**	**Head Size**	**Level of study**	**No of patients (hip)**	**Follow-up**	**Dislocation**	**Acetabular cup fixation**	**Revision rate**
[75]	<36mm	Prospective, Longitudinal and Observational	237 hips	12-Oct	NA	0.40%	0.80%
[76]	36mm	Prospective	22 hips	13-Apr	18%	6.50%	4%
[77]	28mm	Retrospective	48	82 months	NA	8.30%	2.5-30%
	32mm		47	56 months	1.40%	8.50%	
	36mm		49	28 months	0.70%	40.80%	
[78]	32mm	Retrospective cohort study	101, 443	6.6 yrs	2.84%	4-8%	4.70%
[79]	32mm	Retrospective	296 hips	2 yrs	3.38%	0.66%	NA
[80]	>28mm	Prospective	40 hips	15 months	1.40%	NA	NA
[81]	36mm	Retrospective Cohort study	65 hips	2 yrs	3.00%	1.50%	NA
[82]	36mm/40mm	Retrospective	370 patients	12 months	1.10%	8%	10%c
